# Docosahexaenoic Acid Induces Cell Death in Human Non-Small Cell Lung Cancer Cells by Repressing mTOR via AMPK Activation and PI3K/Akt Inhibition

**DOI:** 10.1155/2015/239764

**Published:** 2015-08-03

**Authors:** Nayeong Kim, Soyeon Jeong, Kaipeng Jing, Soyeon Shin, Soyeon Kim, Jun-Young Heo, Gi-Ryang Kweon, Seung-Kiel Park, Tong Wu, Jong-Il Park, Kyu Lim

**Affiliations:** ^1^Department of Biochemistry, School of Medicine, Chungnam National University, Daejeon 301-747, Republic of Korea; ^2^Infection Signaling Network Research Center, School of Medicine, Chungnam National University, Daejeon 301-747, Republic of Korea; ^3^Stem Cell Research and Cellular Therapy Center, Affiliated Hospital of Guangdong Medical College, Zhanjiang 524001, China; ^4^Department of Pathology and Laboratory Medicine, Tulane University School of Medicine, New Orleans, LA 70112, USA; ^5^Cancer Research Institute, School of Medicine, Chungnam National University, Daejeon 301-747, Republic of Korea

## Abstract

The anticancer properties and mechanism of action of omega-3 polyunsaturated fatty acids (*ω*3-PUFAs) have been demonstrated in several cancers; however, the mechanism in lung cancer remains unclear. Here, we show that docosahexaenoic acid (DHA), a *ω*3-PUFA, induced apoptosis and autophagy in non-small cell lung cancer (NSCLC) cells. DHA-induced cell death was accompanied by AMP-activated protein kinase (AMPK) activation and inactivated phosphatidylinositol 3-kinase (PI3K)/Akt/mammalian target of rapamycin (mTOR) signaling. Knocking down AMPK and overexpressing Akt increased mTOR activity and attenuated DHA-induced cell death, suggesting that DHA induces cell death via AMPK- and Akt-regulated mTOR inactivation. This was confirmed in Fat-1 transgenic mice, which produce *ω*3-PUFAs. Lewis lung cancer (LLC) tumor cells implanted into Fat-1 mice showed slower growth, lower phospho-Akt levels, and higher levels of apoptosis and autophagy than cells implanted into wild-type mice. Taken together, these data suggest that DHA-induced apoptosis and autophagy in NSCLC cells are associated with AMPK activation and PI3K/Akt inhibition, which in turn lead to suppression of mTOR; thus *ω*3-PUFAs may be utilized as potential therapeutic agents for NSCLC treatment.

## 1. Introduction

Lung cancer is the main cause of cancer-related death worldwide. According to the latest statistics from the United States National Cancer Institute, it is estimated that 224,210 Americans will be diagnosed with lung cancer in 2014 [1]. There are two types of lung cancer, namely, small-cell lung cancer (SCLC) and non-small-cell lung cancer (NSCLC), with more than 80% of lung cancer cases being NSCLC [2]. Because NSCLC is much less sensitive to chemotherapy than SCLC [3], a new approach for treating NSCLC is required.

Autophagy is a lysosome-associated degradation process that is characterized by the formation of double-membraned autophagosomes, which encapsulate cytoplasmic constituents [4–6]. The degraded components can then be used for energy production and other cellular processes [7]. Autophagy-related (Atg) genes, such as Atg12 and Atg5, are key molecules in regulation of autophagy. Atg12 constitutively associates with Atg5 to form Atg12-Atg5 conjugate, which is essential for the formation of lapidated microtubule-associated light chain 3 (LC3-II, a mammalian homolog of Atg8-II) and autophagosomes [8, 9]. The most potent inhibitor of autophagy is mammalian target of rapamycin (mTOR), which acts upstream of Atg proteins to regulate cell growth/proliferation, survival, protein and lipid synthesis, lysosome biogenesis, and cytoskeletal organization [10–12]. mTOR is activated by Akt, a downstream product of phosphatidylinositol 3-kinase (PI3K), whereas the major negative regulator of mTOR is 5′ AMP-activated protein kinase (AMPK), which regulates intracellular energy status by sensing the AMP/ATP ratio [13–15]. mTOR utilizes p70 ribosomal S6 kinases 1 (S6K1) and eIF4E-binding protein 1 (4E-BP1) as substrates [16]. Inhibiting mTOR not only blocks the phosphorylation of S6K1 and 4E-BP1, but also induces autophagy [16–18].

Docosahexaenoic acid (DHA), an omega-3 polyunsaturated fatty acid (*ω*3-PUFA), induces apoptosis in tumor cells by regulating several key signaling mediators, including Wnt/*β*-catenin [19], p53-regulated activator protein 1 [20], and mitogen-activated protein kinase [21, 22]. In addition, we have previously reported that DHA simultaneously induces apoptosis and autophagy in human cervical cancer as well as prostate cancer cells, and this process involves mTOR repression [23, 24]. Despite several studies describing that the anti-lung cancer activity of DHA may be dependent on its proapoptosis effect [21, 25–27], it is still unclear whether it also induces autophagy* in vitro* and* in vivo*.

Here, we examined the mechanism(s) underlying DHA-induced cell death in human NSCLC cells. The results showed that DHA reduced cell viability and induced both apoptosis and autophagy. Moreover, DHA-induced cell death was associated with AKT-mTOR signaling inhibition and AMPK activation. Similarly, lung cancer cells implanted into Fat-1 transgenic mice exhibited higher levels of apoptosis, a higher autophagy index, and lower levels of phospho-AKT than cells implanted into wild-type mice. Taken together, these data show that DHA induces apoptosis and autophagy through AKT-mTOR signaling inhibition and AMPK activation, suggesting that *ω*3-PUFAs may be a potential treatment for human NSCLC.

## 2. Materials and Methods

### 2.1. Chemicals and Antibodies

DHA (Cayman Chemical, Ann Arbor, MI, USA; dissolved in absolute ethanol), chloroquine (CQ, Sigma, ST Louis, MO, USA), Bafilomycin A1 (Tocris, Bristol, UK; dissolved in phosphate buffered saline (Sigma)), and rapamycin (Tocris; dissolved in dimethyl sulfoxide (DMSO; Sigma)) were stored at −20°C before use.

The following antibodies were used in this study: PI3K (p85), Akt, phospho-Akt (Ser473), phosphophosphatase and tensin homolog deleted on chromosome 10 (PTEN), AMPK, phospho-AMPK (Thr172), phospho-mTOR (Ser2448), phospho-S6K1 (Thr389), Atg5, Atg7, 4E-BP1, and LC3B (all Cell Signaling Technology, Beverly, MA, USA) and poly(ADP-ribose) polymerase- (PARP-) 1/2 (H-250), phospho-Akt (Thr308), p27 (C-19), and actin (I-19)-R (all Santa Cruz, CA, USA). Goat anti-rabbit and goat anti-mouse secondary antibodies were purchased from Calbiochem (Billerica, MA, USA).

### 2.2. Cells Lines and Cultures

Human NSCLC A549 cells and H1299 cells were purchased from American Type Cell Culture Collection (Rockville, MD, USA) and maintained in Dulbecco's Modified Eagle's Medium (DMEM; GIBCO, Grand Island, NY, USA) supplemented with 10% heat-inactivated fetal bovine serum (FBS; GIBCO), penicillin, and streptomycin (GIBCO) in a humidified 5% CO_2_ atmosphere at 37°C.

### 2.3. Cell Viability Assay

Cell viability was determined using thiazolyl blue tetrazolium bromide (MTT; Sigma). Cells were seeded into 96-well plates (7 × 10^3^ per well) and incubated for 18 h at 37°C to allow adherence. The cells were then incubated with serum-free medium for 24 h and then treated with DHA for another 24 h. The cells were then incubated with MTT for 2 h and the formazan products dissolved in DMSO. Absorbance was assayed at 570 nm in a spectrophotometer (Thermo Fisher Scientific, Waltham, MA, USA).

### 2.4. Western Blot Analysis

Western blot analysis was performed as described previously [28]. Briefly, cell lysates (30 *μ*g) were resolved by 6–15% sodium dodecyl sulfate-polyacrylamide gels and then transferred to polyvinylidene difluoride membranes (Millipore, Billerica, MA, USA). The membranes were then blocked in 5% (w/v) skimmed-milk for 1 h, followed by incubation with appropriate primary antibodies (diluted 1 : 1000–1 : 5000) overnight at 4°C. Bound antibodies were detected with peroxidase-conjugated goat anti-rabbit or goat anti-mouse secondary antibodies and the blots developed using enhanced chemiluminescence (Millipore).

### 2.5. Apoptosis Analysis

Apoptosis was measured using a terminal deoxynucleotidyl transferase dUTP nick end labeling (TUNEL) assay, flow cytometry, and an Annexin V assay as described previously [23, 29].

### 2.6. Immunofluorescence Analysis

Cells were grown to 70% confluence in growth medium for 18 h before infection with recombinant adenoviruses expressing GFP-tagged LC3 (GFP-LC3, a gift from Professor Chang Deok Kim, Chungnam National University, Korea). DHA was then added to the infected cell cultures for 24 h. The cells were observed by an Olympus iX70 fluorescence microscope.

### 2.7. Small Interfering RNAs (siRNAs) and Transfection

siRNAs targeting Atg5 and Atg7 were purchased from Invitrogen (Camarillo, CA, USA). A nontargeting control siRNA was purchased from Bioneer (Daejeon, Korea). Cells (4.5 × 10^5^ cells in a 100 mm dish) were transfected with 50 nM of siRNA for 36–48 h using Lipofectamine 2000 reagent (Invitrogen) according to the manufacturer's instructions. The cells were then incubated for 24 h with serum-free medium and then treated with DHA. The siRNA sequences were as follows: nontargeting control siRNA;* 5*′*-ACG UGA CAC GUU CGG AGA AUU-3*′; Atg5,* 5*′*-AUC CCA UCC AGA GUU GCU UGU GAU C-3*′; Atg7,* 5*′*-CCA AGG AUG GUG AAC CUC AGU GAA U-3*; and AMPK* 5*′*-GGU UGG CAA ACA UGA AUU GdTdT-3*′.

For the transfection of expression vectors, cells were grown to 70–80% confluence and then switched to serum-free medium for 2–4 h before being transiently transfected with HA-tagged myr-Akt1 (Akt-wt) and HA-tagged kinase-dead (K179M) dominant negative Akt1 (Akt-dd) (kindly provided by Dr. Incheol Shin; Hanyang University, Korea) using Lipofectamine 2000 reagent (Invitrogen) according to the manufacturer's instructions. After 12–18 h, the cells were subjected to different treatments. Control transfections were carried out using an empty pcDNA3 vector.

### 2.8. Tumor Xenograft Study

Transgenic Fat-1 C57BL/6 mice were provided by Dr. J. X. Kang (Harvard University, USA). Control C57BL/6 mice were purchased from Central Lab Animal Inc. (Seoul, Korea). The control and Fat-1 C57BL/6 mice were kept under specific pathogen-free conditions and received care according to the guidelines of the Institutional Animal Care and Use Committee of Chungnam National University. The Fat-1 C57BL/6 mice (*n* = 5) used in this study were heterozygous, male, and 6 weeks old at the time of the experiments. Each mouse was subcutaneously injected with Lewis lung cancer (LLC) cells (3 × 10^6^ in 100 *μ*L serum-free DMEM) on day 0. The tumor size was measured every other day using calipers for 10 days. Tumor size was calculated as length × wide and volume was calculated as 0.5 × length × (wide)^2^.

### 2.9. Immunohistochemistry

After deparaffinization and antigen retrieval, the implanted tumor cell tissues were blocked with Dako protein block (Dako, Glostrup, Denmark); stained with rabbit anti-phospho-Akt (Ser473; 1 : 250), rabbit anti-phospho-AMPK (1 : 250), and rabbit anti-LC3B (1 : 250) primary antibodies followed by TRITC-conjugated anti-rabbit IgG (1 : 500); and then counterstained with DAPI. Stained tissues were examined under an Olympus iX70 fluorescence microscope using the DP Controller software. Images from two separate channels were merged.

### 2.10. Statistical Analysis

Statistical analyses were done as recommended by an independent analyst. These included the unpaired Student's *t*-test. All values are expressed as mean ± SD, and statistical significance was accepted for *P* values of <0.05. ^*∗*^, ^*∗∗*^, and ^*∗∗∗*^ mean *P* < 0.05, *P* < 0.01, and *P* < 0.001, respectively.

## 3. Results and Discussion

### 3.1. DHA Reduces Cell Viability and Induces Apoptosis in Human NSCLC Cells

**Figure 1:**
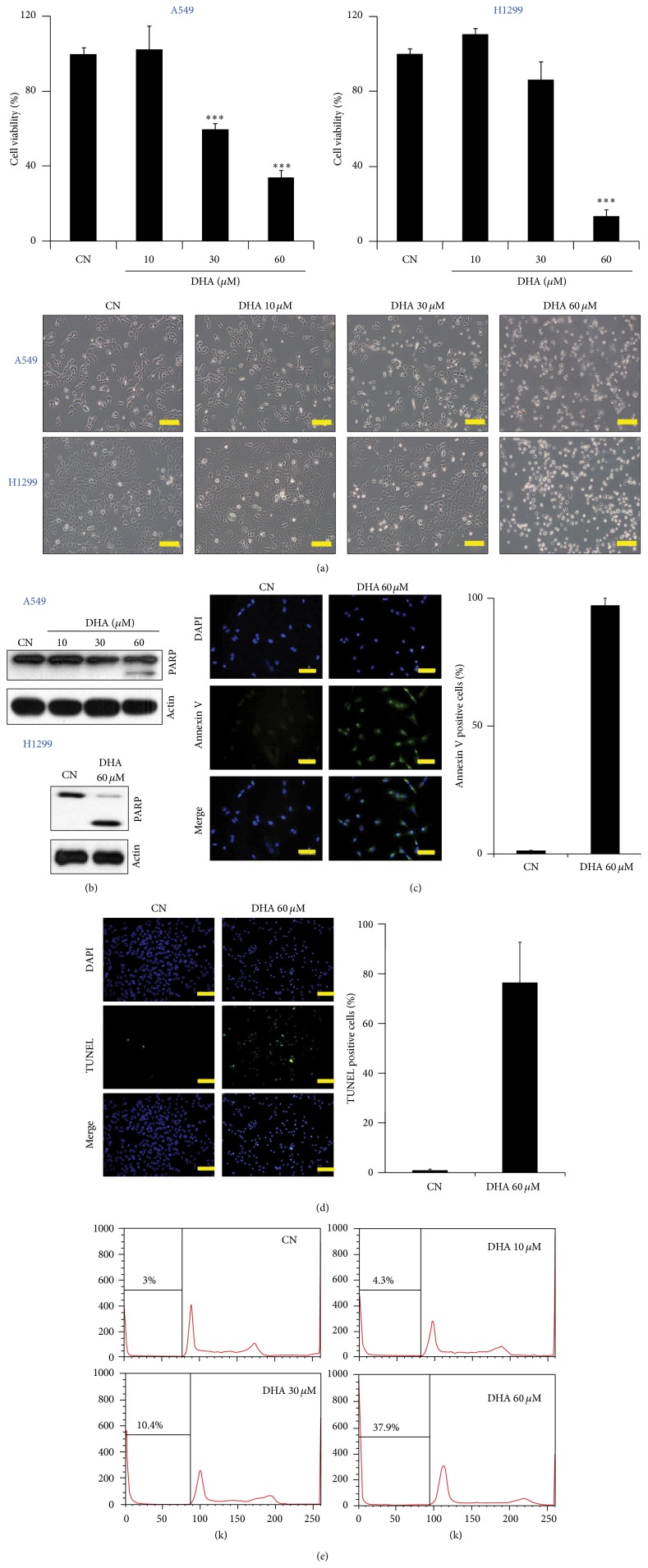
DHA inhibits cell viability and induces apoptosis in human cancer cells. (a) Upper panel: DHA reduces the viability of A549 and H1299 cells in a dose-dependent manner. Cells were exposed to the indicated doses of DHA for 24 h and the cell viability was measured in an MTT assay. Each bar represents the mean of three determinations. Each experiment was repeated three times. ^*∗∗∗*^ *P* < 0.001. Lower panel: representative images of A549 and H1299 cells treated with DHA for 24 h (scale bar: 200 *μ*m). (b) DHA induces apoptosis. A549 (upper panel) and H1299 (lower panel) cells were incubated with the indicated doses of DHA for 24 h. The cells were then harvested and western blot analysis was performed with anti-PARP and anti-actin antibodies. (c) Left panel: evaluation of apoptosis by Annexin V staining. Green staining represents Annexin V-positive (apoptotic) cells (scale bar: 50 *μ*m). Right panel: unfixed A549 cells were treated with FITC-Annexin V and then subjected to flow cytometry to examine changes in the plasma membrane. (d) DHA increases the number of TUNEL-positive cells. A549 cells were plated in a 12-well plate containing glass coverslips and then treated with 60 *μ*M DHA for 6 h. Following treatment, apoptosis was detected using the DeadEnd Fluorometric TUNEL System. Left panel: representative fluorescence microscopy images (scale bar: 200 *μ*m). Right panel: the percentage of TUNEL-positive cells in the presence or absence of DHA was expressed relative to the total number of DAPI-stained nuclei. TUNEL-positive cells were counted in three different fields and the numbers averaged. (e) DHA increases the number of NSCLC cells in the sub-G_1_ phase. A549 cells were seeded and treated with the indicated doses of DHA for 24 h. The cell-cycle distribution of DHA-treated cells was analyzed by flow cytometry as described in Section 2. Data were analyzed using FlowJo software.

It has been shown previously that DHA induces apoptotic cell death in several types of cancer cells [27, 30–32]. To examine whether DHA induces apoptosis in human NSCLC cells, we tested the effect of DHA on the viability of A549 and H1299 cells* in vitro*. DHA reduced the viability of both cell lines (Figure 1(a), upper). Moreover, when observed under a light microscope, DHA-treated cells appeared shrunken, rounded, and detached from the culture dishes (Figure 1(a), lower), characteristics suggestive of apoptosis.

To investigate whether this observed DHA-induced reduction in cell viability was actually due to apoptosis, we first examined the cleavage of PARP, an apoptotic marker. DHA treatment led to an increase in the levels of cleaved PARP in both A549 and H1299 cells (Figure 1(b)). Furthermore, DHA treatment caused a marked increase in the number of Annexin V-positive cells (Figure 1(c)), which is another early apoptotic indicator [33]. We next performed a TUNEL assay and cell-cycle analysis to look for nuclear DNA-strand breaks and hypodiploid DNA formation because both are increased during apoptosis [34]. DHA treatment increased the number of TUNEL-positive A549 cells (Figure 1(d)) and the percentage of A549 cells in the sub-G1 phase (Figure 1(e)). Taken together, these data indicate that DHA induces apoptosis in NSCLC cells.

### 3.2. DHA Triggers Autophagy as a Prerequisite for Apoptotic Cell Death

**Figure 2:**
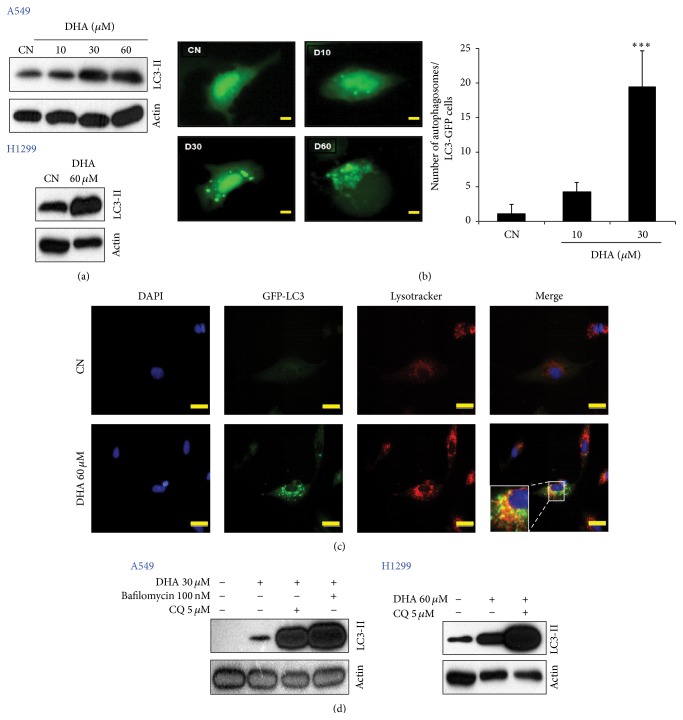
DHA induces autophagy. (a) DHA increased the expression of LC3-II in a dose-dependent manner. A549 (upper panel) and H1299 (lower panel) cells were exposed to the indicated doses of DHA for 24 h. Protein lysates were then prepared, separated in SDS polyacrylamide gels, and immunoblotted with antibodies against LC3-II and actin. (b) Formation of GFP-LC3 puncta in DHA-treated NSCLC cells. A549 cells were transfected with a GFP-LC3 plasmid and then exposed to the indicated doses of DHA for another 24 h before counterstaining with DAPI. Left panel: representative fluorescence microscopy images are shown (scale bar: 2 *μ*m). Right: the number of autophagosomes was quantified as the number of GFP-LC3 puncta per transfected cell. Data are expressed as the mean ± SD of ten determinations (each in two separate experiments). ^*∗∗∗*^ *P* < 0.001. (c) DHA activates autophagic flux in NSCLC cells. Cells were transfected with the GFP-LC3 expression vector using Lipofectamine 2000 reagent for 17 h and then treated with 60 *μ*M DHA for another 4 h. DHA-treated cells were then stained with Lysotracker dye. Representative fluorescence microscopy images are shown. Data are expressed as the mean ± SD of five determinations (each in three separate experiments) (scale bar: 10 *μ*m). (d) DHA-induced autophagy increases NSCLC cell death. A549 (left) and H1299 (right) cells were incubated for 1 h in the presence or absence of the indicated doses of Bafilomycin and CQ before incubation with indicated doses of DHA for 24 h. Cell lysates were prepared and examined by western blotting.

Apoptosis and autophagy are highly interconnected [35], and our previous studies showed that DHA activates both of these cascades simultaneously in cervical and prostate cancer cells [23, 24]. To determine whether autophagy is also involved in DHA-induced NSCLC cell death, we initially measured the expression LC3-II (the membrane-bound lipidated form of LC3), a biomarker of autophagic initiation [36]. Western blot analysis revealed that DHA caused a remarkable increase in LC3-II expression (Figure 2(a)). Similarly, DHA led to a dose-dependent increase in the number of GFP-LC3 puncta per virally infected A549 cell (Figure 2(b)). It is known that during autophagy, autophagosomes fuse with lysosomes in which both LC3-II and the cargo are degraded [23]. To examine whether DHA interferes with autophagic flux, we therefore next examined the colocalization of lipidated LC3-II with lysosomes using Lysotracker dye. DHA treatment led to a marked increase in the colocalization of lipidated LC3-II with Lysotracker compared with that in control cells (Figure 2(c)), suggesting that DHA does not block autophagosome-lysosome fusion. To further confirm this, A549 cells were exposed to DHA in the absence or presence of CQ and Bafilomycin A1 (inhibitors of lysosomal acidification) and LC3-II levels were analyzed by immunoblotting (Figure 2(d)). We found that the DHA-induced increase in LC3-II expression was further increased by CQ and Bafilomycin A1. Taken together, these results indicate that autophagy is activated and may play a role in DHA-induced cell death.

**Figure 3:**
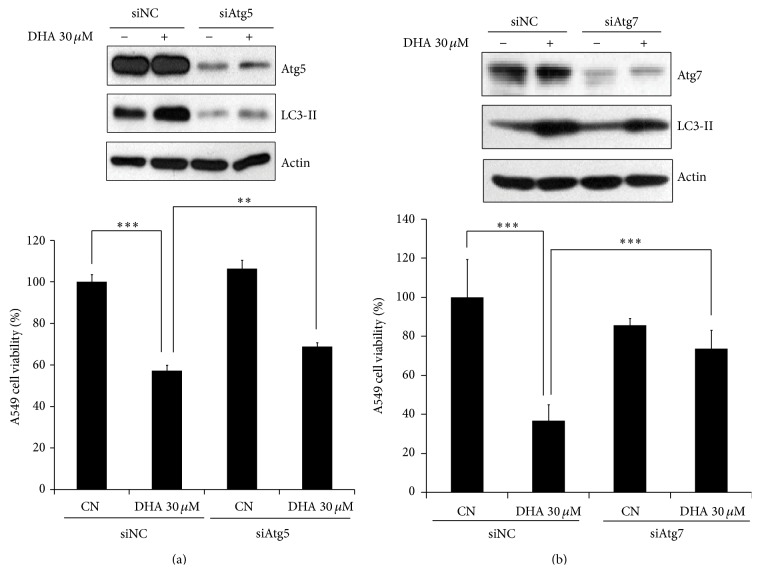
DHA-induced autophagy is required for apoptotic cell death. The indicated cancer cell lines were treated with nontargeting control small interfering RNA (siNC) or siRNAs specific for autophagy-related Atg5 (a) and Atg7 (b). At 18 h after transfection, cells were incubated with the indicated doses of DHA for 24 h. Next, cells were harvested and subjected to western blot analysis with the following antibodies: Atg5, Atg7, LC3-II, and actin (upper panel). Cell viability was measured in an MTT assay (lower panel). ^*∗∗*^ *P* < 0.01 and ^*∗∗∗*^ *P* < 0.001. Data are representative of three independent experiments, all with similar results.

To unveil the relationship between DHA-autophagy and DHA-apoptotic cell death, we used siRNAs to knock down two essential autophagy gene products, Atg5 and Atg7. Although silencing of Atg5 and Atg7 had no effect on the viability of A549 cells, it strongly suppressed DHA-induced autophagy and apoptosis as evidenced by a reduction in LC3-II and viability (Figures 3(a) and 3(b)). These results imply that autophagy, at least partially, contributes to DHA-induced apoptotic cell death.

Autophagy has been shown to be essential for cell survival under certain stressful conditions. For example, hypoxia and the anticancer drug, Nelfinavir, induce autophagy by inactivating the growth factor receptors and by suppressing Akt signaling [37, 38], both of which play a positive role in cell survival; however, our data indicate that DHA induces autophagy, which enhances cell death. This observation is in line with other reports showing that Clioquinol and Rhabdastrellic acid-A promote cell death in hepatocellular carcinoma, lung adenocarcinoma, myeloma, and leukemia cells lines by inducing autophagy [36, 39]. Although the exact mechanism by which autophagy contributes to DHA-induced apoptosis in NSCLC cells is still unclear, it is known that autophagy can promote cell death by selectively eliminating vital components, such as mitochondria and peroxisomes [40]. We have reported that DHA-induced apoptosis is associated with mitochondrial damage [24, 31]; therefore, it is possible that DHA-induced autophagy is triggered by eliminating components that are essential for survival, such as mitochondria.

### 3.3. DHA-Mediated Downregulation of mTOR Signaling Is Associated with Autophagy Induction

**Figure 4:**
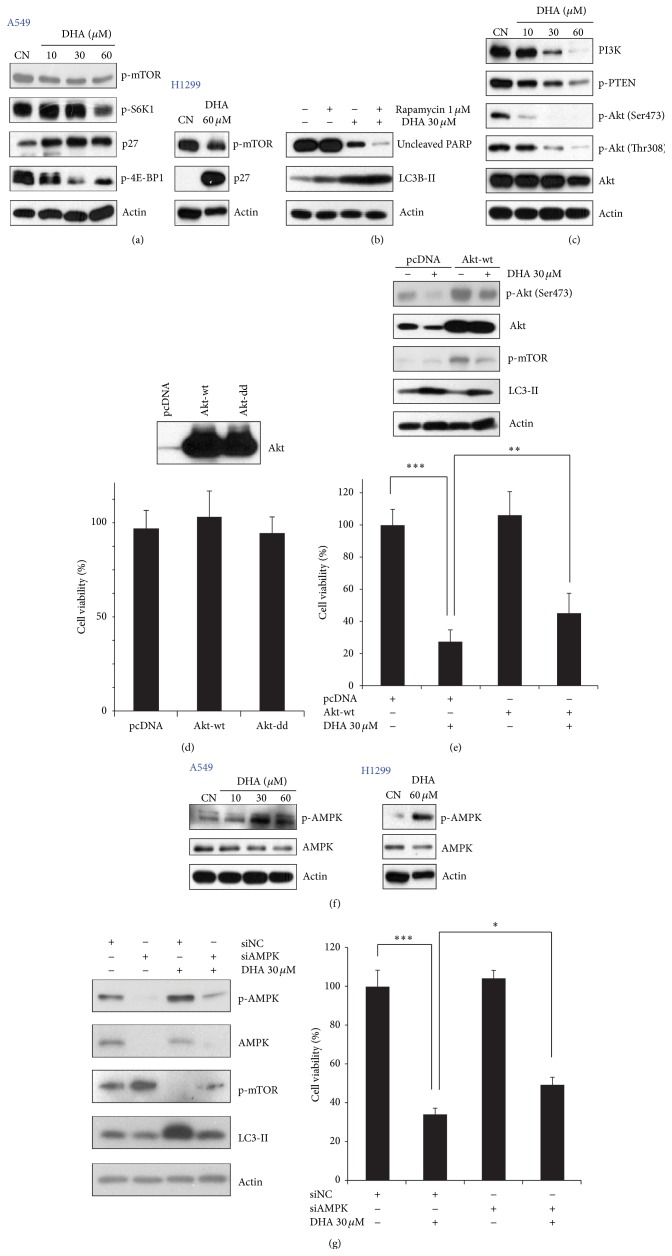
DHA-mediated downregulation of mTOR signaling is related to the induction of autophagy. (a) DHA downregulated mTOR signaling in a dose-dependent manner. A549 (left panel) and H1299 (right panel) cells were incubated with the indicated doses of DHA for 24 h and then subjected to western blot analysis with antibodies against phospho-mTOR, phospho-S6K1, p27, 4E-BP1, and actin. (b) Rapamycin accelerated autophagy and cell death by inhibiting mTOR. A549 cells were incubated for 1 h with or without 1 *μ*M rapamycin before incubation for 24 h with 30 *μ*M DHA. Cell lysates were prepared and examined by western blotting. (c) DHA reduces PI3K/Akt signaling pathway. Western blotting with antibodies against phosphatidylinositol 3-kinase (PI3K)/Akt signaling molecules showed that DHA downregulates PI3K/Akt signaling in a dose-dependent manner. (d)-(e) Expression of Akt-wt partially rescued DHA-induced NSCLC cell death. pcDNA and a Akt-wt vector were transfected into cells using Lipofectamine 2000 reagent for 12 h. The cells were then exposed to 30 *μ*M for another 24 h. Cell viability was examined in an MTT assay ((d) and (e), lower panel) and the cell lysates were analyzed by western blotting with antibodies against phospho-Akt, Akt, phospho-mTOR, and actin ((e), upper panel). ^*∗∗∗*^ *P* < 0.001. (f) DHA treatment led to a dose-dependent increase in phospho-AMPK levels. A549 (left panel) and H1299 (right panel) cells were treated with indicated doses of DHA for 24 h and cell lysates were examined by western blotting. (g) siAMPK reduced DHA-induced autophagy and inhibited cell death in NSCLC cells by upregulating mTOR signaling. A549 cells were transfected with a siNC or siAMPK and then exposed to 30 *μ*M DHA for 24 h. Left panel: western blot analysis of phospho-AMPK, AMPK, phospho-mTOR, LC3-II, and actin expression. Right panel: cell viability was measured in an MTT assay. ^*∗*^ *P* < 0.05 and ^*∗∗∗*^ *P* < 0.001.

mTOR is the key negative regulator of autophagy [4]. To examine whether mTOR inhibition is involved in DHA-induced autophagy, we investigated the expression of mTOR signaling-related molecules in NSCLC cells after DHA treatment. DHA reduced the levels of phospho-mTOR in both A549 (Figure 4(a), left panel) and H1299 (Figure 4(a), right panel) cells, indicating that the activity of mTOR is repressed by DHA. Consistent with this, the levels of mTOR's two readout molecules, phospho-S6K1 and phospho-4E-BP1, were also found to be decreased in A549 cells. Meanwhile, increases in p27 (whose activation is indicative of mTOR inhibition) [23] were observed in DHA-treated A549 and H1299 cells (Figure 4(a)), suggesting that DHA indeed suppresses the mTOR signaling pathway. Next, to confirm the role of mTOR in DHA-induced cell death, we pretreated A549 cells with rapamycin followed by DHA. We found that DHA-induced decreases in uncleaved PARP and increases in LC3-II expression in A549 cells were enhanced by pretreatment with rapamycin (Figure 4(b)). These results imply that DHA-induced autophagy and apoptosis are associated with mTOR inhibition.

mTOR is directly linked to PI3K/Akt signaling [41], and the PI3K/Akt/mTOR signaling pathway plays an important role in cell proliferation and survival [42]. We then asked whether PI3K/Akt is associated with DHA-induced mTOR inactivation. To test this, we examined the expression of PI3K/Akt signaling molecules and found that DHA induced a marked reduction in PI3K, Akt, and phospho-PTEN (a negative regulator of the PI3K) (Figure 4(c)). Next, to obtain evidence for the interconnection between decreased PI3K/Akt signaling and DHA-induced cell death, we overexpressed Akt in A549 cells before DHA treatment (overexpression in itself had no significant effect on the cell viability of A549 cells) (Figure 4(d)). DHA treatment led to a reduction in cell viability and increased the levels of phospho-mTOR and LC3-II; however, these phenomena were partially reversed by Akt overexpression (Figure 4(e)). These data suggest that DHA-induced cell death is also associated with Akt inhibition in NSCLC cells. Evidence suggests that DHA can disrupt the association between lipid rafts and epidermal growth factor receptor (EGFR), leading to inactivation of EGFR and its downstream PI3K/Akt signaling in lung cancer cells [43]. Accordingly, it is reasonable to speculate that DNA might inhibit the PI3K/Akt signaling pathway by disrupting EGFR phosphorylation and its association with lipid rafts; however, further studies are needed to investigate whether this is the mechanism underlying DHA-mediated Akt inactivation.

In addition to Akt, another key mediator of mTOR is AMPK, which has been demonstrated to negatively regulate mTOR activity [15]. We sought subsequently to determine whether DHA-induced mTOR inactivation involves AMPK in NSCLC cells. To this end, we first measured the levels of AMPK in DHA-treatment A549 and H1299 cells. DHA treatment led to increased AMPK activity in both A549 and H1299 cells, as evidenced by the promoted expression levels of phospho-AMPK (Figure 4(f)). Importantly, knockdown of AMPK not only rescued DHA-induced mTOR inactivation, but also inhibited DHA-induced autophagy and cell death in A549 cells (Figure 4(g)). These results demonstrated that DHA-induced autophagy mediated by mTOR inhibition, at least partly, attributes to AMPK activation. Previously, endoplasmic reticulum (ER) stress has been shown to elevate AMPK levels, leading to the upregulation of Ca^2+^-CaMKK*β* signaling and autophagy-related genes such as Atg5 and Atg12 [44]. We found that the levels of phosphoeukaryotic translation initiation factor-2*α*, which reflects ER stress, increased in DHA-treated A549 cells in a time-dependent manner (data not shown). Thus, although the exact mechanism underlying DHA-mediated AMPK activation remains unknown, it is possible that AMPK activation in DHA-treated cells is induced by the activation of Ca^2+^-CaMKK*β* and autophagy-related genes via ER stress.

### 3.4. *ω*3-PUFAs Suppress Tumor Growth* In Vivo* by Inhibiting the Expression of Phospho-Akt and Phospho-AMPK, Thereby Inducing Apoptosis and Autophagy

**Figure 5:**
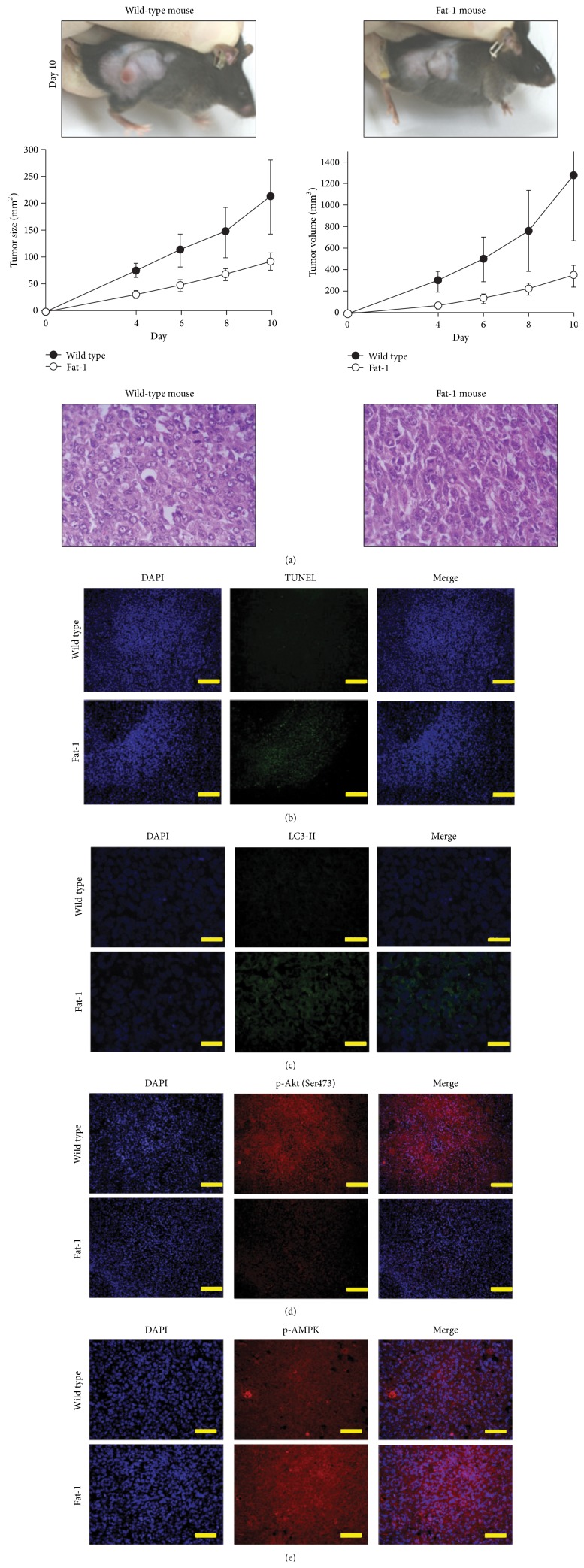
*ω*3-PUFAs suppress tumor growth* in vivo* by inhibiting phospho-Akt and phospho-AMPK, thereby inducing apoptosis and autophagy. (a) Effect of *ω*3-PUFA on tumorigenicity. Upper panel: LLC cells (3 × 10^6^ cells) were injected subcutaneously into the flanks of wild-type and Fat-1 transgenic mice. Tumor size and volume were monitored every other day for 10 days. Tumor size and volume (middle) were calculated as described in Section 2. Lower panel: hematoxylin and eosin (H&E) staining. ((b)–(e)) Representative fluorescence images showing the TUNEL assay results (b), LC3-II (c), phospho-Akt (Ser473) (d), and phospho-AMPK (e). Fluorescently stained tissues were observed under a fluorescence microscope using DP Controller software (Olympus) for image acquisition. Scale bars: 200 *μ*m.

The* in vitro* results presented above show that DHA reduced the viability of human NSCLC cells. We next examined the effect of DHA on tumor formation and growth in Fat-1 transgenic mice. Fat-1 transgenic mice express *ω*3-desaturase and thus produce higher levels of *ω*3-PUFAs than wild-type (WT) mice [45]. The mouse origined NSCLC, LLC cells were subcutaneously injected into WT mice and Fat-1 transgenic mice, and tumor size and volume were measured. We found that both the size and volume of the tumors in Fat-1 transgenic mice were markedly lower than those in WT mice (Figure 5(a)), suggesting that *ω*3-PUFAs suppress the growth of NSCLC cells* in vivo*. To verify whether apoptosis and autophagy played a role in this process, we performed TUNEL assays and indirect immunofluorescence assays on tumor tissue sections to examine apoptosis and autophagy levels, respectively. The number of TUNEL-positive cells and the number of LC3-II puncta were higher in Fat-1 tumors than in WT tumors (Figures 5(b) and 5(c)), indicating that both apoptosis and autophagy played a role in inhibiting tumor growth in Fat-1 transgenic mice. Next, to determine whether PI3K/Akt and AMPK signaling were responsible for the increased levels of apoptosis and autophagy, we examined the levels of Akt and AMPK in tumor tissues by immunohistochemistry. As shown in Figures 5(d) and 5(e), the levels of phospho-Akt (Ser473) decreased and the levels of phospho-AMPK increased in Fat-1 tumor tissues. Together, these data demonstrate that *ω*3-PUFAs induce apoptosis and autophagy* in vivo* by regulating AMPK and PI3K/Akt signaling.

## 4. Conclusions

**Figure 6:**
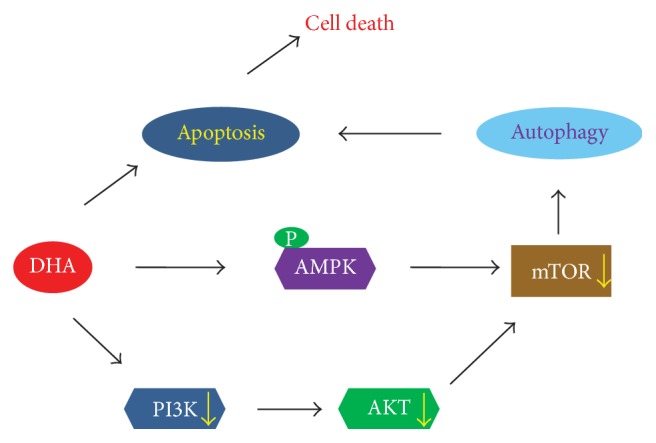
Schematic model of DHA-induced apoptosis and autophagy in NSCLC cells. DHA-induced autophagy and apoptosis in lung cancer cells are triggered by inhibition of mTOR activation via AMPK activation and PI3K/Akt inhibition.

In conclusion, we describe for the first time that DHA triggers autophagy and apoptosis in NSCLC cells, which simultaneously promotes cell death. Our results indicate that the DHA-induced autophagy and apoptosis are controlled by repressing mTOR through AMPK activation and PI3K/Akt inhibition (Figure 6). These data suggest that DHA may represent a potentially useful reagent for treating human NSCLC in clinical settings.
